# A case report of reversible posterior encephalopathy syndrome with intracranial hemorrhage in a child

**DOI:** 10.1097/MD.0000000000025266

**Published:** 2021-03-26

**Authors:** Xiaoqian Chen, Weixue Zhu, Suhua Jiang

**Affiliations:** Department of Pediatrics, First People's Hospital of Foshan, Foshan, Guangdong, PR China.

**Keywords:** Brain damage, Child, Intracranial hemorrhage, Reversible posterior encephalopathy syndrome

## Abstract

**Introduction:**

The objective is to analyze the clinical diagnosis and treatment of children with rescindable posterior encephalopathy syndrome (PRES) and intracranial hemorrhage (ICH) to improve the pediatrician's understanding of PRES combined with ICH in children.

**Patient concerns and Diagnosis:**

After liver transplantation, the patient developed symptoms of epilepsy and coma. Meanwhile, massive necrosis of acute cerebral infarction and small hemorrhage was observed in the left cerebellar hemisphere and left occipital lobe, respectively. The above symptoms were initially diagnosed as PRES.

**Interventions and outcomes:**

After adjusting the anti-rejection drug regimen, it was found that the child's neurological symptoms were relieved, and the limb motor function gradually recovered during follow-up. Imaging examination showed significant improvement on abnormal signals in brain.

**Conclusion:**

In general, children with PRES may further develop ICH and contribute to a poor prognosis. Early diagnosis, detection of risk factors and timely adjustment of medication regimen are the keys to prevent irreversible brain damage.

## 1 Introduction

Posterior reversible encephalopathy syndrome (PRES), as a complication, was first reported in 1996, and was related to fluid overload, hypertension, or immunosuppression. The clinical manifestations of PRES include convulsion, headache, reduced vision, changes in mental state. Critical PRES can develop into intracranial hemorrhage (ICH), herniation, seizures, and other serious life-threatening complications, with an ultimate mortality rate as high as 16%.^[1,2]^ So far there are few reports on children's PRES combined with ICH in China. Hence, we report a case of PRES combined with ICH after liver transplantation in our pediatric intensive care unit, and summarized its clinical and imaging information to deepen the understanding of the disease, and further guide clinical diagnosis and treatment.

## 2 Case presentation

On July 2, 2018, our patient, a 4.5 years old boy with blood type AB Rh^+^, was identified with congenital biliary atresia for more than 4 years and a pale complexion for 1 week. The vital signs on admission were as follows: body temperature: 36.8 °C; pulse, 100 times/min; respiratory rate, 25 times/min; weight: 14 kg (< −2SD); length, 90 cm; and blood pressure, 103/74 (84) mmHg (blood pressure> 95th percentile). An abdominal examination revealed obvious abdominal distension and abdominal varices. A subsequent ultrasonic examination showed splenomegaly and no edema in the lower extremities.

Peripheral blood analysis revealed the following results: white blood cell count, 1.68 × 10^9^ /L; red blood cell count, 1.83 × 10^12^ /L; hemoglobin, 34 g/L; platelet count, 22 × 10^9^ /L; and fibrinogen, 2.13 g/L. This patient underwent biliary atresia surgery in 2014. On July 5, 2018, he underwent allogeneic orthotopic liver transplantation under general anesthesia followed by the treatment of Meroperem and anticoagulation with low-molecular-weight heparin after surgery. Subsequently, the patient received the Atomolan, Minolol and Ulinastatin for the anti-rejection and anti-inflammatory treatment.

## 3 Diagnostic assessment

**Figure 1:**
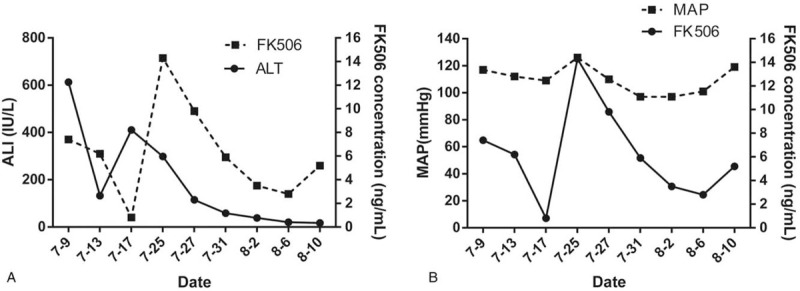
(A) Changes in plasma levels of liver function alanine aminotransferase (ALT) and tacrolimus (FK506) after liver transplantation; (B) Changes in MAP and tacrolimus (FK506) blood drug concentration after liver transplantation. MAP = mean arterial pressure.

After transplantation, the serum level of AST, as an important liver function indicator, gradually decreased to the normal range. The post-operative blood concentration of tacrolimus is shown in the Figure 1A. On the seventh postoperative day, the child developed seizures accompanied by the following other symptoms: a twitch in the corner of the mouth, frequent teeth grinding, restlessness, light coma with glasgow coma scale score of 7-point next day, no speech, weakened right limb activity, neck resistance and bilateral pupils were unequal in size with 4 mm on the left and 2.5 mm on the right. Bilateral Pap sign was positive. Blood pressure fluctuation of child was 86–163/64–111 (85–127) mm Hg and the blood concentration of tacrolimus and fibrinogen were 6.2 ng/L and 0.67 g/L, respectively.

The relationship between blood drug concentration and mean arterial pressure is shown in Figure 1B. On the same day, the computed tomography scan showed that the patient had high-density shadows in the right frontal parietal-ventricular body. It was considered that the patient had a high possibility of cerebral hemorrhage ruptured into the ventricular system with patchy low-density reduction area on the left frontal lobe and parietal occipital lobe right parietal lobe and corpus callosum, acute cerebral hemorrhage ruptured into the ventricles, while there was small hematocele in the posterior horn of both lateral ventricles, with the midline shifted slightly towards the left.

**Figure 2:**
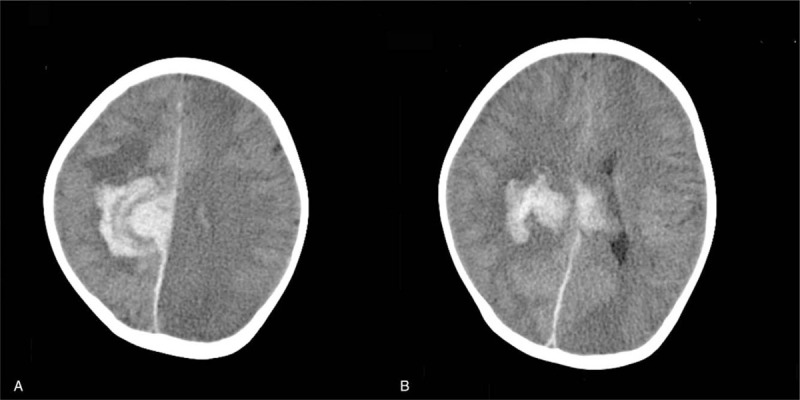
CT on the 7th postoperative day. Note: There are massive high-thickness tracker in the anterior parietal lobe-ventricular body on the right side, and low-density edema gumshoe on the surrounding brain parenchyma. The space-occupying effect is obvious. The right lateral ventricle and the midline structure are compressed and shifted to the left. Both sides High-density shadows were seen in the later horn of the lateral ventricle. The left forward lobe and parietal occipital part show flaky low-density reduction areas.

Magnetic resonance imaging (MRI) in Figure 2 showed right parietal lobe and the corpus callosum, acute cerebral hemorrhage ruptured into the ventricles, while there was a small hematocele in the posterior horn of both lateral ventricles, with the midline slightly left. Moreover, massive acute cerebral infarction was observed in the left cerebellar hemisphere with predominantly cortical infarction, suggesting the occurrence of PRES. In addition, there was a small hemorrhage in the left occipital lobe, and susceptibility weighted imaging showed a relatively increased number of small blood vessels on the surface of the bilateral cerebral hemispheres. Based on the above findings, the child was preliminarily diagnosed with PRES.

## 4 Therapeutic intervention

**Figure 3:**
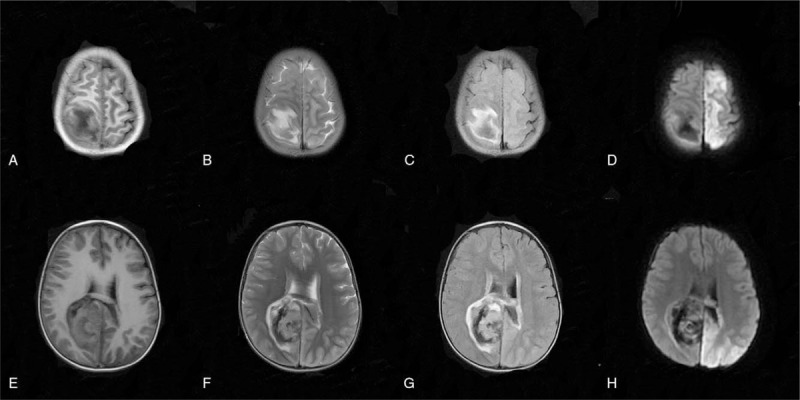
Head MRI on the 7th postoperative day. Note: A and E are T1 weighted image (T1WI), B and F are T2 weighted image (T2WI), C and G are fluid attenuated inversion recovery (FLAIR), and D and H are diffusion-weighted imaging (DWI). MRI = magnetic resonance imaging.

Significant ICH was observed on the right side (Fig. 3). The patient further received the intravenous injection of mannitol combined with concentrated sodium dehydration and nifedipine, along with gradual reduction of heparin dosage to improve blood coagulation, fibrinogen supplementation. Finally, this patient received antiepileptic treatment with topiramate combined with levetiracetam. Given that tacrolimus predisposes to PRES, its dose was reduced to 0.5 mg q12 h. After the above treatment for 7 consecutive days, oral captopril was used instead of intravenous nifedipine, and the blood pressure fluctuated between 97–130/76–104 (81–102) mm Hg. After 1 more week of treatment, the patient's clinical status was as follows: lethargic with glasgow coma scale score 10-point, intermittent restlessness, less teeth grinding, improved response and bilateral pupils equal in size (both about 3 mm in diameter), sensitive on the left side and slightly dull on the right side. Muscle strength of 4 extremities were grade I and muscle tension was slightly higher. Bilateral Pap sign was still positive. The blood level of transaminase gradually increased when the blood concentration of tacrolimus decreased to 0.8 ng/mL. Considering the rejection reaction, the amount of tacrolimus was gradually increased to 1 mg q12 h again on the 16th postoperative day. On the 34th day after surgery, the child's blood pressure increased again, fluctuating between 121–142/73–108 (88–119) mm Hg, followed by irritability, frequent mouth twitching and teeth grinding, neck resistance, and bilateral pupils ranging in size with the left pupil of 3 to 4 mm and sensitive to light, the right pupil of 2.5 to 3.5 mm and insensitive to light. Muscle strength of 4 extremities were grade I and muscle tension was slightly higher, specially, obvious on the right side. The large and small joints were slightly stiff. Both sides had positive Pap sign. Blood concentration of tacrolimus and fibrinogen were 5.2 ng/L and 2.37 g/L, respectively. Reexamination of the head computed tomography revealed the following results: hemorrhage in the right frontoparietal lobe - corpus callosum was absorbed more than the previous image with decreased density; the bilateral parietal occipital lobe and the large corpus callosum was ischemic with infarct softening manifestations and the density decreased compared with anterior slice; uneven thickness of cerebral arteries were improved compared with anterior slice; right middle cerebral artery M2 was decreased significantly with distal branches; right posterior cerebral artery P1 segment was slightly narrowed.

**Figure 4:**
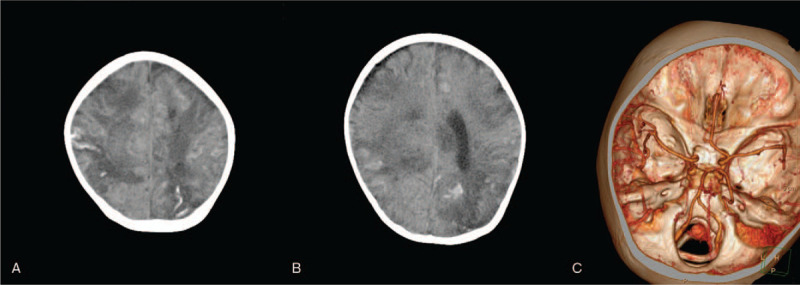
Head CT on day 35. Note: A and E are T1WI, B1 and B2 are T2WI, B and F are FLAIR, C and G are FLAIR, and D and H are DWI. DWI = diffusion-weighted imaging. FLAIR = fluid attenuated inversion recovery, T1WI = T1 weighted image, T2WI = T2 weighted image.

The arteries were still small (Fig. 4) and the MRI showed that the right parietal lobe and corpus callosum hemorrhage were smaller than before; the bilateral cerebral hemisphere cortex and cortical infarction were changed, and the left frontotemporal lobe showed local acute stage; bilateral basal ganglia and thalamus nuclei were considered ischemic deficiencies, especially acute changes on the left side; a small hemorrhage foci and a little subarachnoid hemorrhage on the left and right frontal lobe, respectively, were observed.

**Figure 5:**
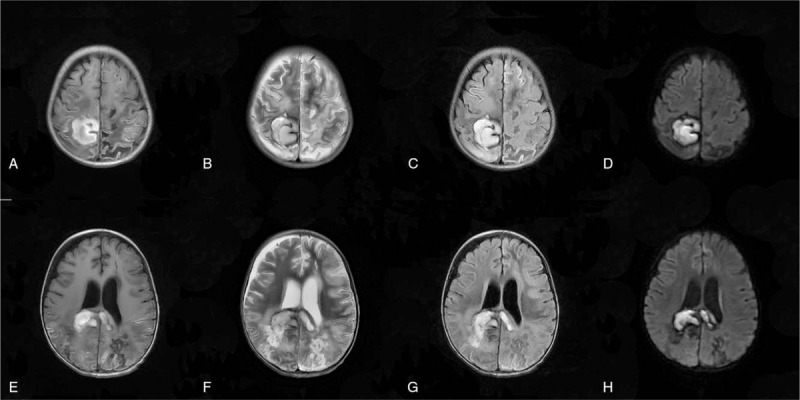
Head MRI on day 35 after surgery. Note: A and E are T1WI, B1 and B2 are T2WI, B and F are FLAIR, C and G are FLAIR, and D and H are DWI. DWI = diffusion-weighted imaging, FLAIR = fluid attenuated inversion recovery, MRI = magnetic resonance imaging, T1WI = T1 weighted image, T2WI = T2 weighted image.

There was a small hematocele in the posterior horns of both lateral ventricles (Fig. 5). After increasing the dose of tacrolimus, the patient developed aggravated neurological symptoms. Consequently, we stopped tacrolimus, and switch to sirolimus (0.5 mg qd) to resist rejection and gradually increased to 1 mg qd. The blood concentration of sirolimus fluctuated between 3.0 to 5.2 ng/L, and the hepatic AST gradually decreased to normal level. The patient continuously received subsequent treatment of antiepileptic and nutritional support, and the physical rehabilitation training was also started.

## 5 Follow-up and outcomes

**Figure 6:**
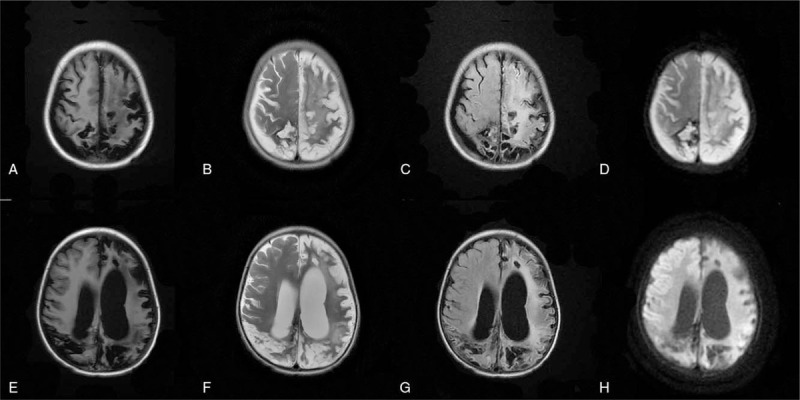
Head MRI 5 months after surgery. Note: A and E are T1WI, B and F are T2WI, C and G are FLAIR, and D and H are DWI. DWI = diffusion-weighted imaging, FLAIR = fluid attenuated inversion recovery, MRI = magnetic resonance imaging, T1WI = T1 weighted image, T2WI = T2 weighted image.

A 5-month postoperative review of the head MRI showed that the hematoma on the right parietal lobe and the corpus callosum were significantly absorbed, and the original brain edema was alleviated (Fig. 6). Subcortical changes were considered as cortical infarction. The original left frontotemporal lobe was part of old cerebral infarction, and the hemorrhage of left hemorrhagic foci of the occipital lobe and subarachnoid were both absorbed. Meanwhile, a little blood absorption was observed in the posterior horn of the hemiventricle. The supraventricular ventricles were obviously enlarged with occurrence of hydrocephalus, and a small amount of subventricular effusion was also observed on the top of the right forehead. During follow-up to August 2019, the child's right limb muscle strength was basically recovered, the left limb muscle strength was still poor, and the activity was limited.

## 6 Discussion

PRES is a relatively rare severe central nervous system syndrome caused by multiple predisposing factors, and its imaging features present diversity, so its pathophysiology remains quite controversial.^[3]^ Clinical manifestations of PRES include headache, nausea/vomiting, seizures, loss of consciousness, and decreased visual acuity. Typical imaging findings are reversible posterior cerebral cortex and subcortical angioedema.^[4]^ The most common cause of PRES is renal insufficiency caused by various reasons, followed by hematological malignancies, various organs and tissues damage caused by immunosuppressants (such as cyclosporin A, tacrolimus) and cytotoxic drugs in organs and tissues transplantation. However, there are few literatures reported the related researches of PRES combined with ICH in children and only some reports indicated that the incidence of ICH in patients with PRES is close to 20%.^[5,6]^

In our patient, multiple factors of irritability, hypermyotension, and convulsions during liver transplantation after immunosuppressive anti-rejection therapy were associated with PRES. The main clinical symptoms were irritability and convulsions, and the symptoms were relieved along with the decrease of drug dose. Therefore, this patient was initially diagnosed with PRES, but also presented with imaging findings of ICH. The exact pathogenesis of PRES is not yet clear. At present, there are 2 understandings:

(1) Cerebrovascular regulatory dysfunction and destruction of the blood-brain barrier integrity cause the development of PRES;
(2) Under normal circumstances, when the systolic blood pressure increases, the intracranial blood vessels can regulate themselves and constrict vessels.

Nevertheless, the systolic blood pressure of PRES patients suddenly exceeds the degree of tolerance of the intracranial vessels and the blood-brain barrier is destroyed, resulting in increased hydrostatic pressure. It can leak from blood vessels into brain tissue and cause cerebral edema.^[4]^ The mechanisms of complication of ICH in PRES patients are not fully elucidated and may include:

(1) hypertension causing vascular rupture;
(2) damage to vascular endothelial cells, causing damage to the blood-brain barrier;
(3) abnormal coagulation such as thrombocytopenia;
(4) anticoagulant drugs use increases the risk of bleeding;
(5) cerebral hemorrhage caused by other coexisting diseases of the body.^[7]^

The mechanism of ICH in patients with PRES has not been fully elucidated and may include:

(1) hypertension causing vascular rupture;
(2) damage to vascular endothelial cells, resulting in damage to the blood-brain barrier;
(3) coagulation abnormalities such as thrombocytopenia;
(4) increased risk of bleeding with anticoagulant use; and
(5) cerebral hemorrhage caused by other coexisting diseases of the body.

The high-risk factors of PRES combined with ICH in this patient may include side effects of immunosuppressive agents, abnormal coagulation function and hypertension. The nervous system symptoms such as high muscle tone and convulsions in this patient were significantly relieved after reducing the dose of tacrolimus, a new type of calcineurin inhibitor (CNI), which is widely used in organ transplantation and nephrotic syndrome. However, these symptoms get worse when the dose of tacrolimus is increased again. These results suggested that high dose of tacrolimus may be the most important factor to induce PRES. Previous studies demonstrated that CNI can bind to immunophilin, and then its blood drug concentration will decrease.^[8]^ In addition, tacrolimus can hinder the construction of nitric oxide in the brain, which in turn causes cerebral vasoconstriction, apoptosis and blood-brain barrier changes. The hepatic cytochrome P450 metabolism system and bile acid excretion system were also affected by post-transplant liver injury, which may be associated with an amplified risk of neurotoxicity.^[9,10]^ Sirolimus is often used as an alternative to replace CNI with less neurotoxicity and nephrotoxicity after organ transplantation.^[11]^ Preoperative impaired splenic function in children leads to excessive platelet retention and destruction, and thrombocytopenia after liver transplantation also affects coagulation.

Liver transplantation leads to the reduction of hepatic protein synthesis function, and the level of vitamin K-dependent coagulation factors is obviously reduced, which leads to the reduction of blood coagulation function. In addition, preoperative blood pressure indicated poor vascular elasticity. Blood pressure was high after surgery, which may not only cause PRES, but may also lead to intracranial vascular rupture and bleeding.

It has been reported that older age is 1 of the high-risk factors for intracranial loss after surgery.^[12]^ More than 90% of pediatric liver transplant recipients in China are less than 1 year of age, and children have thin blood vessels and a higher risk of vascular complications, so stronger anticoagulation treatment is required after surgery.^[13]^ However, this patient was older (4 years old) and the vessel diameter and fragility were closer to adults. Therefore, this patient is more prone to intracranial loss, and further studies are necessary to investigate the need for initial anticoagulant therapy in older children in the future.

The time required for the relief of neurological symptoms in children is relatively long, and the rate of limb recovery is significantly inconsistent. Previous studies reported complete recovery in 89.3% of PRES patients treated with CNI.^[14]^ It is reported that the incidence of neurological diseases after liver transplantation in children was 15% to 35%, and children are more likely to fully recover than adults.^[15,16]^ After switching from tacrolimus to sirolimus, the blood drug concentration in patient was 5.2 ng/mL 1 week later, and the neurological symptoms also gradually improved. After rehabilitation therapy, the patient's left limb muscle tension and activity returned to normal, but the right limb muscle tension was high and the movement was still limited while suggesting that the left limb may be more reversible due to less ICH.

In recent years, remarkable achievements have been made in pediatric liver transplantation, which has become a standardized treatment and held promise for survival of many children with end-stage liver disease. Children after liver transplantation mostly suffer neurological complications.^[17]^ Reports of reversible posterior encephalopathy in children have gradually increased and have attracted the attention of pediatricians. However, few cases of PRES combined with ICH have been reported. The key to treat PRES is to the efficient control of blood pressure, anticonvulsant therapy, and removal or reduction of predisposing factors.^[18]^ Furthermore, the key to the treatment of ICH is hemostasis and improved coagulation. The disadvantage of this case is that the perioperative blood pressure was not promptly vigilant. Meanwhile, postoperative anticoagulation requires close monitoring of coagulation function and timely adjustment of the anticoagulation plan. An alternative immunosuppressant, tacrolimus, should be considered when the neurological symptoms once developed. PRES can cause complex ICH, herniation, and status epilepticus, resulting in poor patient prognosis.^[17]^ Therefore, organ transplantation should be performed in collaboration with critically ill children and neurologists to ensure rapid and accurate diagnosis and appropriate management of neurological complications.

## Author contributions

**Conceptualization:** Suhua Jiang.

**Data curation:** Xiaoqian Chen.

**Formal analysis:** Xiaoqian Chen, Weixue Zhu.

**Supervision:** Suhua Jiang.

**Validation:** Xiaoqian Chen.

**Visualization:** Xiaoqian Chen.

**Writing – original draft:** Xiaoqian Chen.

**Writing – review & editing:** Suhua Jiang, Weixue Zhu.

## References

[1] Legriel S Schraub O Azoulay E. Determinants of recovery from severe posterior reversible encephalopathy syndrome. PLoS One 2012;7:e44534. 23024751 10.1371/journal.pone.0044534 PMC3443081

[2] Flynn JT Kaelber DC Baker-Smith CM. Clinical practice guideline for screening and management of high blood pressure in children and adolescents. Pediatrics 2017;140:e20171904. 28827377 10.1542/peds.2017-1904

[3] Wu X Shen Q. 4 cases of children with reversible posterior encephalopathy syndrome and literature review (Chinese). Zhejiang Medicine 2017;39:298–300.

[4] Mu Q Liu G. Research status and progress of reversible posterior encephalopathy syndrome in children (Chinese). Medical Recapitulate 2015;21:1053–5.

[5] Sharma A Whitesell RT Moran KJ. Imaging pattern of intracranial hemorrhage in the setting of posterior reversible encephalopathy syndrome. Neuroradiology 2010;52:855–63. 19956935 10.1007/s00234-009-0632-6

[6] Hefzy HM Bartynski WS Boardman JF. Hemorrhage in posterior reversible encephalopathy syndrome: imaging and clinical features. AJNR Am J Neuroradiol 2009;30:1371–9. 19386731 10.3174/ajnr.A1588 PMC7051550

[7] Wen Y He W Huang Q. Posterior reversible encephalopathy syndrome complicated with intracranial hemorrhage: clinical analysis of 5 patients literature review (Chinese). Chin J Nerv Ment Dis 2016;42:537–41.

[8] Diekmann F Budde K Oppenheimer F. Predictors of success in conversion from calcineurin inhibitor to sirolimus in chronic allograft dysfunction. Am J Transplant 2004;4:1869–75. 15476488 10.1111/j.1600-6143.2004.00590.x

[9] Hammerstrom AE Howell J Gulbis A. Tacrolimus-associated posterior reversible encephalopathy syndrome in hematopoietic allogeneic stem cell transplantation. Am J Hematol 2013;88:301–5. 23460378 10.1002/ajh.23402

[10] Lee YJ Yum MS Kim EH. Risk factors for neurological complications and their correlation with survival following pediatric liver transplantation. Pediatr Transplant 2014;18:177–84. 24372703 10.1111/petr.12218

[11] Tian P Ao J Li N. Technical specification for clinical application of organ transplant immunosuppressants (2019 edition) (Chinese). Organ Transplantation 2019;10:7–20.

[12] Ling L He X Zeng J. Early intracranial hemorrhage after liver transplantation. Chin J Nerv Ment Dis 2007;33:209–12.

[13] Sun L. Pediatric liver transplantation (Chinese). Chin J Appl Clin Pediatr 2017;32:818–20.

[14] Song T Rao Z Tan Q. Calcineurin inhibitors associated posterior reversible encephalopathy syndrome in solid organ transplantation. Medicine 2016;95:e3173. 27057842 10.1097/MD.0000000000003173 PMC4998758

[15] Piñero F Cheang Y Mendizabal M. Incidence, risk factors, and outcomes related with neurological events after liver transplantation in adult and pediatric recipients. Pediatr Transplant 2018;22:e13159. 29417691 10.1111/petr.13159

[16] Ghosh PS Hupertz V Ghosh D. Neurological complications following pediatric liver transplant. J Pediatr Gastroenterol Nutr 2012;54:540–6. 22167017 10.1097/MPG.0b013e3182407de3

[17] Servillo G Bifulco F De Robertis E. Posterior reversible encephalopathy syndrome in intensive care medicine. Intensive Care Med 2007;33:230–6. 17119920 10.1007/s00134-006-0459-0

[18] Cordelli DM Masetti R Ricci E. Life-threatening complications of posterior reversible encephalopathy syndrome in children. Eur J Paediatr Neurol 2014;18:632–40. 24814477 10.1016/j.ejpn.2014.04.014

